# Clenbuterol and metformin ameliorate cachexia parameters, but only clenbuterol reduces tumor growth via lipid peroxidation in Walker 256 tumor-bearing rats

**DOI:** 10.1590/1414-431X2024e14060

**Published:** 2025-01-31

**Authors:** L.D.V. Henschel, M.E.R. de Lima, F.C. Fagundes, T. Horlem, M.F. Zazula, K. Naliwaiko, L.C. Fernandes

**Affiliations:** 1 Laboratório de Metabolismo Celular, Departamento de Fisiologia, Setor de Ciências Biológicas, Universidade Federal do Paraná, Curitiba, PR, Brasil; 2 Laboratório de Plasticidade Morfofuncional, Departamento de Biologia Celular e Molecular, Setor de Ciências Biológicas, Universidade Federal do Paraná, Curitiba, PR, Brasil

**Keywords:** Cancer cachexia, Tumor, Clenbuterol, Metformin, Walker 256

## Abstract

Cancer is the second leading cause of death worldwide. Cancer cachexia is a multifactorial catabolic syndrome responsible for almost one third of cancer-related deaths. Drug repurposing has been used in oncological research and drugs like clenbuterol and metformin seem to be reasonable candidates in the context of cancer cachexia, because the former is a β_2_-agonist that stimulates muscle gain and the latter has anti-inflammatory properties. The aim of this study was to assess the effects of a short-term treatment with metformin and clenbuterol, isolated or combined, on tumor growth and cancer cachexia parameters in Walker 256 tumor-bearing rats, a model of cancer cachexia. To this end, Wistar rats were separated into 8 groups and 4 of them were injected with Walker 256 tumor cells (W groups). Control (C) and W groups received the following treatments: metformin (M), clenbuterol (Cb), or metformin combined with clenbuterol (MCb). Body and tumor weight, metabolic parameters, and oxidative damage in the tumor were assessed. Compared to the C group, the W group showed body weight loss, hypoglycemia, hyperlactatemia, and hypertriacylglycerolemia. None of the treatments could reverse body weight loss, although they reversed the alterations of the assessed plasma metabolic parameters. Surprisingly, only clenbuterol alone reduced tumor weight. Hydrogen peroxide production and lipid peroxidation in tumor tissue was increased in this group. In conclusion, metformin and clenbuterol ameliorated metabolic cachexia parameters in Walker tumor-bearing rats, but only clenbuterol reduced the tumor weight, probably, through a lipid peroxidation-dependent cell death.

## Introduction

Cancer is the second leading cause of death worldwide (1). Depending upon tumor type, 20 to 70% of cancer patients may develop a multifactorial catabolic syndrome called cancer cachexia (2). This condition is characterized by ≥5% of body mass loss (3), due to loss of muscle mass and fat mass (4). Cancer cachexia leads to decreased quality of life and reduced overall survival (5). Unfortunately, there is no available treatment for this syndrome (5), and it cannot be fully reversed by nutritional approaches (6). Walker 256 tumor is a model that mimics many key features of cancer cachexia in rats (7).

Currrently, the management of cancer cachexia consists of a multidisciplinary approach, using a combination of drugs, nutraceuticals, and exercise (2). Furthermore, the tendency of the next generation of pharmaceuticals to fight cancer is to aim at many targets of tumor cells simultaneously, using drug repurposing (8). Drug repurposing means using an established drug for a new therapeutic purpose. This approach can reduce costs and time to discover novel therapeutic strategies, while helping to treat some conditions (9).

Metformin (M) is the gold-standard drug for type 2 diabetes mellitus treatment (10). Diabetes patients treated with metformin have reduced risk of cancer development (11,12). Several studies have reported antitumor effects of metformin (11,13) and it has been used in clinical trials (14). Metformin has an antihyperglycemic and lipolytic effect and also can reduce food intake (15). Even though some studies have approached cancer development and metformin as a preventive intervention (16) or as a therapy (17), there is still controversial evidence in the literature about the role of metformin in cancer cachexia. Metformin also has anti-inflammatory properties, because it can inhibit NF-κB (nuclear factor kappa-light-chain-enhancer of activated B cells), reducing expression of pro-inflammatory cytokines (11). In the context of cancer cachexia, this might be useful, as this condition is characterized by systemic inflammation (2). In Walker 256 tumor-bearing rats, metformin reduced tumor weight but did not reverse cancer cachexia-induced weight loss, although it attenuated the inhibition of protein synthesis induced by the tumor (17). On the other hand, using the same model but with a higher dose of metformin, the drug did not decrease tumor weight or mitigate lean and fat mass loss or whole body weight loss (18).

β_2_-agonists have been studied on rodent models of cancer cachexia and seem to attenuate cancer cachexia alone (2,19) or in combination with other drugs (20-22) via muscle mass and protein content increase (19-22). Clenbuterol (Cb) is a bronchodilator drug (23) with a potent anabolic effect on protein synthesis in skeletal muscle (24). A previous study using Walker 256 tumor-bearing rats showed that clenbuterol combined with naproxen and insulin reduced tumor mass and loss of body weight (22). However, whether clenbuterol alone might reduce tumor growth and cancer cachexia is unknown. We are not aware of any study that has investigated the combined treatment of metformin and clenbuterol in a model of cancer cachexia.

Therefore, the aim of this study was to assess the effects of a short-term treatment with metformin and clenbuterol, isolated or combined, on tumor growth and cancer cachexia parameters in Walker 256 tumor-bearing rats. Here, we showed that all of the treatments reversed plasma metabolic parameter alterations induced by the presence of the tumor, although they did not ameliorate body weight loss, and that only clenbuterol alone reduced tumor weight.

## Material and Methods

### Animals

Animal experiments were approved by the Ethical Committee of the Federal University of Parana (CEUA-BIO/UFPR), under protocol 1474. Ninety-day-old Wistar rats were obtained from UFPR's animal facility and maintained under controlled humidity, temperature (23±2°C), and light (12 h light/dark cycle). All of the interventions were made during the light cycle, with water and standard chow *ad libitum*. Animals were weighed every two days after tumor inoculation until euthanasia.

### Experimental design

**Figure 1:**
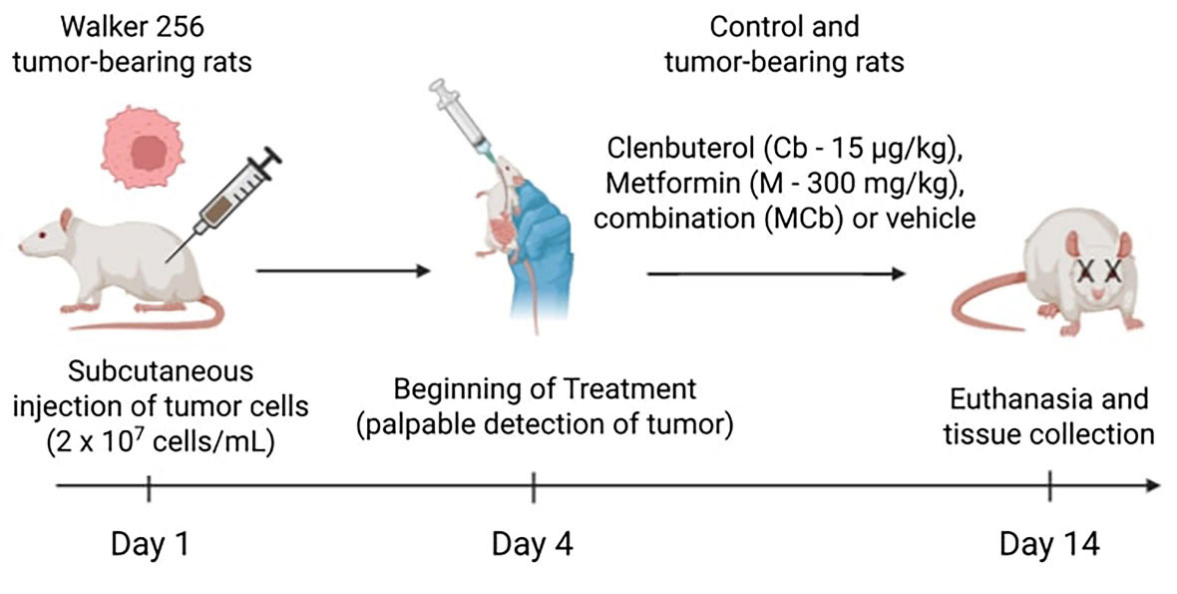
Experimental design. Rats were inoculated with Walker 256 tumor cells on the first day of experiment. On day 4, when the tumor was palpable, the treatment (or vehicle) was administered to control and Walker groups: Cb: clenbuterol, M: metformin, or combined metformin and clenbuterol (MCb). On day 14, rats were euthanized and tissues were collected. The figure was created with BioRender (Canada).

Rats were separated into non-tumor (C) and Walker tumor groups (W). Those belonging to the W groups were inoculated, subcutaneously, with 2×10^7^ Walker 256 tumor cells (22) on the right flank (Figure 1). When the tumor was palpable/detectable (4 days later), the treatment began as previously described (22), in order to mimic what happens with cancer patients. The tumor bearing- and non-tumor-bearing rats were divided into 8 groups: Control (C), Control treated with metformin (CM) (Glifage^®^, Merck, Germany), Control treated with clenbuterol (CCb) (Clenbolic™, Cooper Pharma Limited, England), or metformin and clenbuterol (CMCb). The same treatments were made in the 4 Walker tumor groups (tumor-bearing rats: W, WM, WCb, and WMCb). Treatment was performed every day for 10 days via gavage, using the following dosage: metformin (300 mg/kg) or clenbuterol (15 μg/kg). Animals from the C and W groups also received saline via gavage. The metformin dose used was chosen according to a previous study that used the same model (25), and the clenbuterol dose was selected based on doses utilized in humans as a bronchodilator (23), because of its potency. Its bioavailability varies from 70 to 80% in humans, and its half-life is higher than other drugs from the same class. Therefore, a 10-time lower dose was used compared to our previous study using clenbuterol (22) in order to reduce the risk of adrenergic adverse effects, although we did not find an increase in heart mass in the previous study.

On the 14th day after 12 h of fasting, rats were euthanized by decapitation. This date was chosen based on previous studies using the same model of cancer cachexia to avoid inhumane conditions for the animals (22,26). None of the animals died before this date. Blood was collected, centrifuged at room temperature (Eppendorf, Germany) for 5 min at 2,000 *g*, and plasma was aliquoted and snap-frozen in liquid nitrogen. Fragments of the liver, soleus, and white portion of gastrocnemius muscle were dissected, weighed, and separated for quantification of glycogen content. We chose two different types of muscles in order to investigate whether waste rate depended on the type of fibers. Soleus is a red muscle, with predominantly oxidative metabolism, while the white portion of gastrocnemius muscle has predominantly glycolytic metabolism (27). The tumor was weighed and snap-frozen for further oxidative damage analyses. The weight of the carcass was calculated as: (final body weight - tumor weight) in W groups. Body weight change was calculated as: (final body weight - initial body weight - tumor weight) / initial body weight ×100.

### Plasma analyses

Plasma was used to quantify glucose (Labtest, Brazil), lactate (Bioclin, Brazil), and triacylglycerol (Labtest, Brazil) using biochemical kits, following manufacturer's instructions. The absorbance of the assays was read using a microplate reader (BioTek EL800, USA).

### Glycogen content

The glycogen content was determined as described elsewhere (26). Briefly, the weighted parts of the liver, soleus muscle, and gastrocnemius muscle were quickly immersed into a KOH solution (1 M) at 10 µL/mg tissue. The tissues were digested for 20 min at 70°C. One hundred µL was aliquoted into microtubes and 500 µL acetate buffer at 0.2 M, pH 4.8 and amyloglucosidase were added, followed by 2 h incubation at 37°C. After centrifugation (800 *g*, 5 min, at room temperature), glucose was quantified using a biochemical kit (Labtest, Brazil), following the manufacturer's instructions. The absorbance of the assay was read in a microplate reader (BioTek EL800, USA).

### Oxidative damage analyses

Tumor samples were homogenized in 1 mL of 50 mM Tris-Base Buffer at pH 7.4 and centrifuged for 30 min at 12,000 *g* (Eppendorf) at 4°C. The supernatant was aliquoted for lipid peroxidation, non-protein thiols (NP-SH), and oxygen and nitrogen reactive species assays. The protein concentration was determined by Bradford's method (28).

#### Lipid peroxidation

Lipid peroxidation assay was performed as described elsewhere (29), using the xylenol method. Briefly, 100 μL of the homogenate was added to 900 μL of reaction solution (100 μM xylenol orange, 250 μM Fe^2+^, 25 mM H_2_SO_4_, and 4 mM butylated hydroxytoluene in 90% of methanol). After 30 min of incubation at room temperature, the absorbance was read at 560 nm in the microplate reader (BioTek EL800, USA). The same equipment was utilized for the following analyses. The data are reported in nmoles of hydroperoxide per mg per mL of protein.

#### Non-protein thiols

The measurement of NP-SH was made as described before (30). Briefly, 50 μL trichloroacetic acid (50%) was added to 100 μL of the frozen sample solution, mixed, and centrifuged at 5,000 *g* for 10 min at 4°C. Fifty microliters of the supernatant were added to a microplate, with 230 μL of 0.4 M Tris-Base at pH 8.9 and 20 μL 5,5′-dithiobis-(2-nitrobenzoic acid) solution (0.004 g in 250 μL methanol and 3.75 mL 0.4 M Tris-Base at pH 8.9). After 10 min, at room temperature, the absorbance was verified at 415 nm. The standard curve was made with a serial dilution of glutathione (starting with 1:10 solution of 4 mM). The absorbance was read at 415 nm. Data are reported as μM of NP-SH per mg per mL of protein.

#### Hydrogen peroxide

Measurement of oxygen and nitrogen reactive species followed the protocols previously described (31). To quantify hydrogen peroxide, 100 μL of sample solution was added to 100 μL phenol red in a microplate, which was incubated for 2 h at 37°C, protected from light. At the end, 10 μL of 1 M NaOH was added to each well and the microplate was read at 620 nm. The production of hydrogen peroxide can be seen as oxidation of phenol red. Data are reported as absorbance per mg per mL of protein.

#### Superoxide anion

To measure superoxide anion, 100 μL of sample solution was added to 100 μL of nitroblue tetrazolium (NBT) in a microplate, which was incubated for 2 h at 37°C, protected from light. Superoxide anion reacts with NBT and forms an insoluble compound called formazan. After that, the supernatant was discarded and the microplate was incubated for 10 min with methanol 50% for fixation. Again, the supernatant was discarded and the microplate was kept at 37°C for 15 min. The solubilization of the formazan was made by adding 120 μL of 2 M KOH and 140 μL dimethyl sulfoxide to each well. The microplate was incubated for 30 min at 37°C and the absorbance was read at 560 nm. The data are reported as absorbance per mg per mL of protein.

#### Nitric oxide

Nitric oxide was quantified as nitrite using the Griess reaction. 100 μL of sample solution were added to 200 μL Griess reagent (1:1 of 0.1% naphthyl ethylenediamine chloride dissolved in distilled water and 1% sulfanilamide dissolved in 5% H_3_PO_4_). The color formed by this reaction was read at 550 nm. Results are reported as absorbance per mg per mL of protein.

### Statistical analysis

Statistical analysis was performed using GraphPad Prism^®^ software, version 8 (Dotmatics, USA). Normality of data was confirmed using Kolmogorov-Smirnov test. Data are reported as means±SE. Groups were compared using one-way (for analyses involving only the W groups) or two-way ANOVA (for the other analyses, having as factors: tumor and treatment). We used the *post hoc* Tukey test for multiple comparisons. P-value <0.05 was considered significant.

## Results

**Figure 2:**
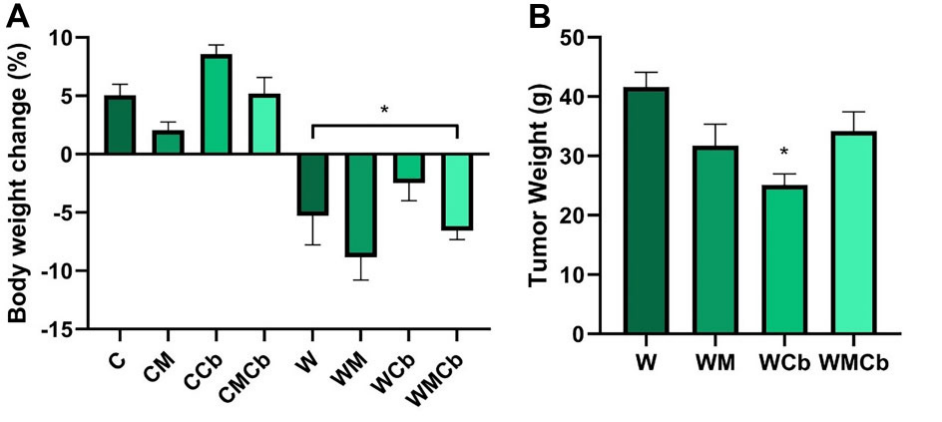
**A**, Body weight change (%) and (**B**) tumor weight (g). Body weight change was calculated as final body weight - initial body weight - tumor weight) / initial body weight × 100. Data are reported as means±SE. *P<0.05 compared to non-tumor-bearing, no treatment control group (two-way ANOVA, *post hoc* Tukey). **B**, *P<0.05 compared to W group (one-way ANOVA, *post hoc* Tukey). C: control group (n=14); CM: control treated with metformin (n=12); CCb: control treated with clenbuterol (n=12); CMCb: control treated with metformin and clenbuterol (n=12); W: tumor-bearing group (n=17); WM: tumor-bearing group treated with metformin (n=7); WCb: tumor-bearing group treated with clenbuterol (n=12); WMCb: tumor-bearing group treated with metformin and clenbuterol (n=8).

Table 1 shows absolute initial and final body weight, and carcass weight. There was no significant difference in absolute body weight. Figure 2A shows body weight change. The tumor factor had a significant effect (Table 2, P<0.0001) on it, as did treatment (Table 2, P=0.0053). However, there was no interaction effect between factors (Table 2, P=0.9800). The non-tumor-bearing control group (C) gained about 5% of body mass during the 14 days, with no effect of treatments. On the other hand, the presence of the tumor caused body weight loss in all W groups (P<0.05 *vs* C) and none of the treatments significantly increased it. In addition, clenbuterol alone reduced tumor weight by almost 40% (Figure 2B, P<0.05 *vs* W). Curiously, adding metformin to clenbuterol treatment disrupted the clenbuterol effect on tumor weight in the WMCb group (P>0.05 *vs* W).

**Table 1:** Initial and final body weight, carcass weight, and tumor weight.

	C	CM	CCb	CMCb
Initial body weight (g)	383±14	354±13	360±10	362±14
Final body weight (g)	401±14	362±13	390±11	380±13
	W	WM	WCb	WMCb
Initial body weight (g)	413±19	348±15	386±19	370±6
Final body weight (g)	433±20	351±20	401±18	380±6
Carcass weight (g)	391±20	319±20	376±18	345±6
Tumor weight (g)	42±3	32±4	25±2	35±3

Data are reported as means±SE. C: control group (n=14); CM: control treated with metformin (n=12); CCb: control treated with clenbuterol (n=12); CMCb: control treated with metformin and clenbuterol (n=12); W: tumor-bearing group (n=17); WM: tumor-bearing group treated with metformin (n=7); WCb: tumor-bearing group treated with clenbuterol (n=12); WMCb: tumor-bearing group treated with metformin and clenbuterol (n=8).

**Table 2:** Effects of individual factors and their interaction.

Parameter	Tumor factor(P-value)	Treatment factor(P-value)	Interaction(P-value)
Body weight change	<0.0001	0.0053	0.9800
Glycemia	0.2371	0.0181	<0.0001
Lactatemia	0.0088	<0.0001	0.0001
Triacylglycerolemia	<0.0001	<0.0001	<0.0001
Hepatic glycogen content	<0.0001	<0.0001	<0.0001
Soleus muscle glycogen content	<0.0001	0.0034	0.0115
Gastrocnemius muscle (white part) glycogen content	<0.0001	0.4140	0.512

P<0.05 was considered statistically significant (two-way ANOVA).

**Figure 3:**
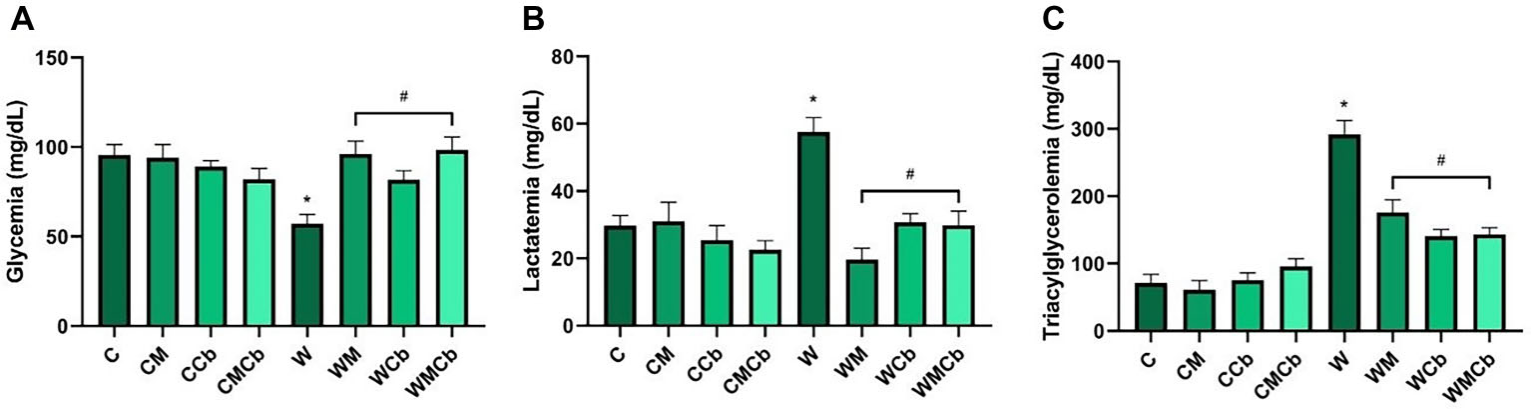
Plasma concentration of (**A**) glucose, (**B**) lactate, and (**C**) triacylglycerol (mg/dL). Data are reported as means±SE. *P<0.05 *vs* C group; ^#^P<0.05 *vs* W group (two-way ANOVA, *post hoc* Tukey). C: control group (n=13); CM: control treated with metformin (n=10); CCb: control treated with clenbuterol (n=8); CMCb: control treated with metformin and clenbuterol (n=8); W: tumor-bearing group (n=14); WM: tumor-bearing group treated with metformin (n=5); WCb: tumor-bearing group treated with clenbuterol (n=6); WMCb: tumor-bearing group treated with metformin and clenbuterol (n=6).

Regarding plasma parameters in the non-tumor-bearing groups, glycemia, lactatemia, and triacylglycerolemia were not affected by different treatments (Figure 3). Tumor presence in the non-treated animals (W) induced hypoglycemia (reduction of almost 40%, Figure 3A, P<0.05 *vs* C), hyperlactatemia (increase of approximately 50%, Figure 3B, P<0.05 *vs* C), and hypertriacylglycerolemia (increase of nearly 400%, Figure 3C, P<0.05 *vs* C). All treatments reversed these blood biochemical parameters of cancer cachexia in Walker 256 tumor-bearing rats (P<0.05 *vs* W), either completely (glycemia and lactatemia, P>0.05 *vs* C) or partially (triacylglycerolemia, P<0.05 *vs* C and *vs* W). In Walker groups, metformin alone increased glycemia by 68% and decreased lactatemia by 33% and triacylglycerolemia by 60% (WM *vs* W). Similarly, clenbuterol alone enhanced glycemia by 42% and reduced lactatemia by 53% and triacylglycerolemia by 48% (WCb *vs* W). The combination of the two drugs (WMCb *vs* W) had similar effects as the isolated drugs on those three parameters, respectively: increase by 58% (glycemia) and reduction by 51% (lactatemia) and by 49% (triacylglycerolemia). Globally, there was no main effect of tumor on glycemia (Table 2, P=0.2371), but there was a treatment and interaction effect on it (Table 2, P<0.05). Regarding lactatemia and triacylglycerolemia, there were effects of tumor and treatment factors and also an interaction effect (Table 2, P<0.05).

**Figure 4:**
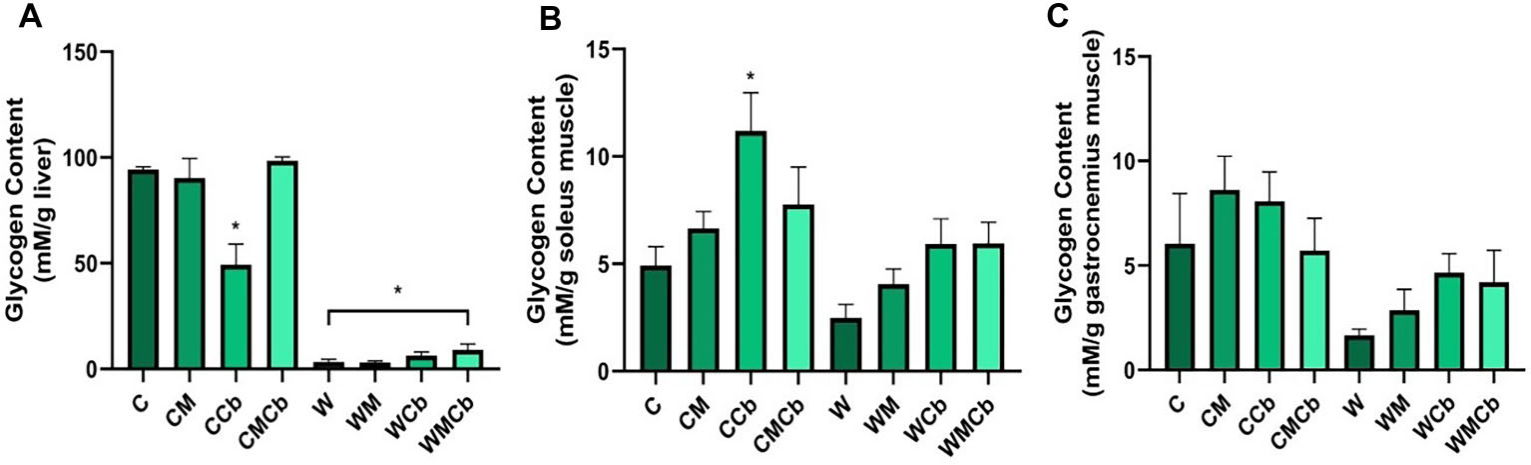
Glycogen content in the (**A**) liver, (**B**) soleus muscle, and (**C**) gastrocnemius muscle (white part). Data are reported as means±SE. *P<0.05 compared to the C group (two-way ANOVA, *post hoc* Tukey). C: control group (n=2); CM: control treated with metformin (n=2); CCb: control treated with clenbuterol (n=4); CMCb: control treated with metformin and clenbuterol (n=2); W: tumor-bearing group (n=4); WM: tumor-bearing group treated with metformin (n=3); WCb: tumor-bearing group treated with clenbuterol (n=4); WMCb: tumor-bearing group treated with metformin and clenbuterol (n=3).

Tumor and treatment had individual effects, as well as an interaction effect on hepatic glycogen content (Table 2, P<0.05). Liver glycogen content (Figure 4A) in all tumor-bearing groups was markedly reduced (P<0.05 *vs* C) and the different treatments did not cause any change (P>0.05 *vs* W). Non-tumor-bearing rats treated with clenbuterol alone (CCb) had lower liver glycogen content compared to the control group (P<0.05 *vs* C), but it was still higher compared to the W groups (P<0.05 *vs* W). There were individual effects as well as an interaction effect of tumor and treatment factors on soleus muscle glycogen content (Table 2, P<0.05). Glycogen content of soleus muscle (Figure 4B) from non-tumor-bearing rats treated with clenbuterol (CCb) was higher compared to the control group (P<0.05 CCb *vs* C). Regarding gastrocnemius muscle glycogen content, there was only a main effect of tumor (Table 2, P<0.05), but not of treatment or their interaction. There was no significant reduction of glycogen content in any muscle induced by tumor presence (Figure 4B and C, P>0.05 W *vs* C), which we attributed to the small sample size of this experiment (n=4 per group). For example, the soleus muscle from the W group showed about half of the glycogen content (2.48±0.62 mM/g tissue) compared to the C group (4.92±0.87 mM/g tissue). Similarly, the WCb (5.93±1.17 mM/g tissue) and the WMCb (5.94±0.99 mM/g tissue) groups had glycogen content twice as high as the W group (2.48±0.62 mM/g tissue) (P>0.05). In gastrocnemius muscle, the W group (1.66±0.29 mM/g tissue) had three-fold lower glycogen content than the C group (6.04±2.4 mM/g tissue). On the other hand, the groups treated with Cb showed an apparent increase in glycogen content (WCb: 4.66±0.91 mM/g tissue; WMCb: 4.2±1.53 mM/g tissue) compared to the W group (1.66±0.29 mM/g tissue, P>0.05).

**Figure 5:**
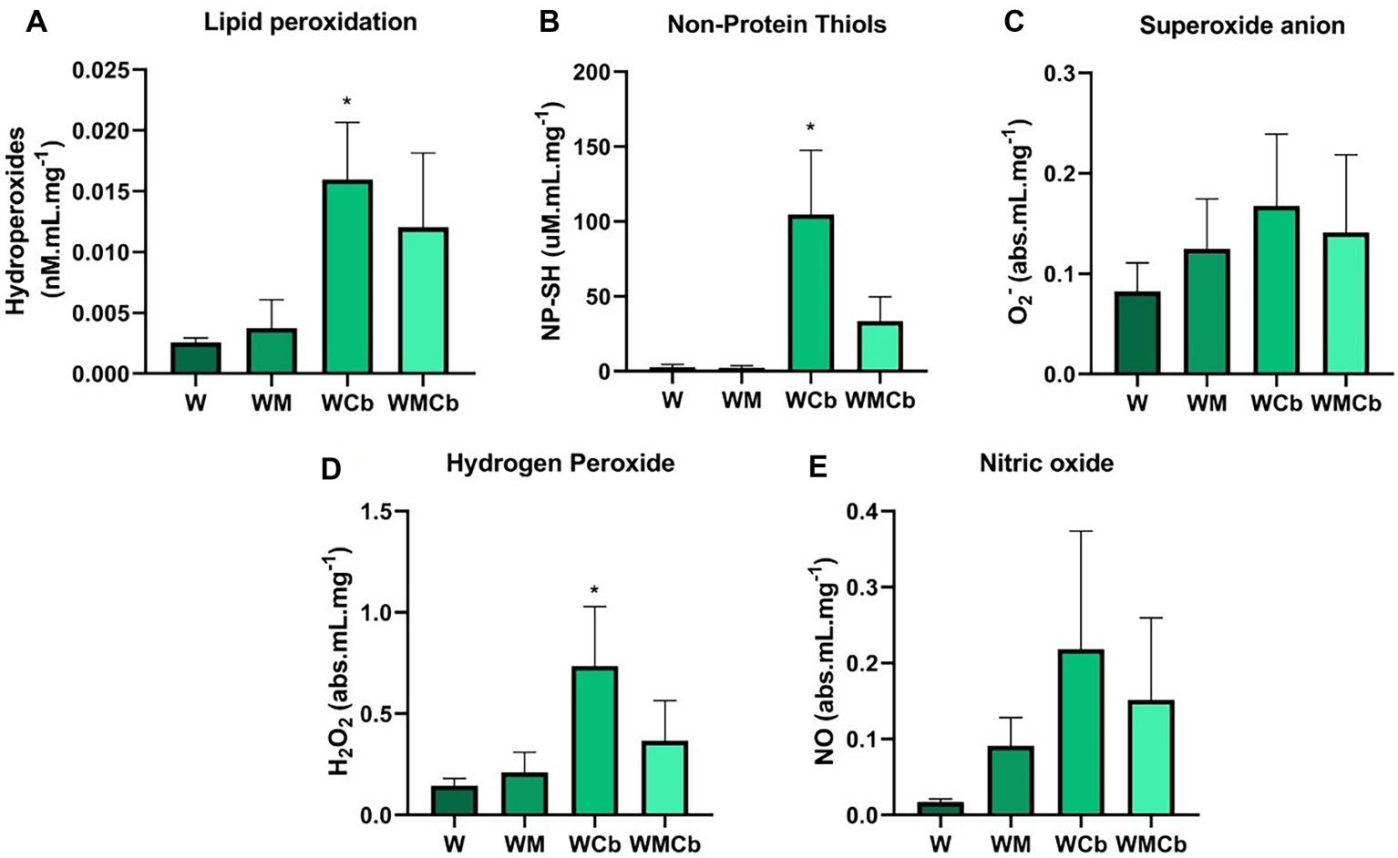
Oxidative damage and reactive species in tumor tissue. **A**, Lipid peroxidation, (**B**) non-protein thiols (NP-SH), (**C**) superoxide anion, (**D**) hydrogen peroxide, and (**E**) nitric oxide. Data are reported as means±SE. *P<0.05 compared to the W group (one-way ANOVA, *post hoc* Tukey). W: tumor-bearing group (n=5); WM: tumor-bearing group treated with metformin (n=6); WCb: tumor-bearing group treated with clenbuterol (n=5); WMCb: tumor-bearing group treated with metformin and clenbuterol (n=4).

Oxidative stress in tumor tissue is reported in Figure 5. Tumors from animals treated with clenbuterol (WCb) showed an increase in lipid peroxidation (Figure 5A), non-protein thiols (Figure 5B), and hydrogen peroxide (Figure 5C) compared to the W group (P<0.05), with no differences in superoxide anion (Figure 5D) and nitric oxide (Figure 5E) between groups.

## Discussion

Cancer cachexia promotes systemic catabolism, body mass loss, hypoglycemia, hyperlactatemia, hypertriacylglycerolemia, and depletion of glycogen storage (2,5,22). Walker 256 tumor is known to mimic these cachectic parameters, being an ideal model to study cancer cachexia in rats (7,22). Walker 256 tumor cells used in this study induced all the alterations in body weight, plasma parameters, and liver glycogen content found in cancer cachexia.

None of the treatments could attenuate body mass loss induced by the tumor. As is well known, any improvement in body weight can impact the quality of life of individuals with cancer cachexia. Cb seemed to reduce the loss of body mass in some rats from the WCb group, perhaps due to its anabolic property (32). It has already been reported that Cb is able to improve protein synthesis via mTOR phosphorylation (33), and glucose tolerance via activation of protein Gs (34). Also, Cb prevents protein catabolism in skeletal and cardiac muscle through inhibition of ATP-ubiquitin-dependent proteolytic pathway in Yoshida AH-130 model of cancer cachexia (35). These effects may justify our anti-cachectic results concerning clenbuterol administration. In the present work, the addition of metformin therapy prevented the effect of Cb. We hypothesized that this may be due to the appetite suppressant effect of metformin (15).

Clenbuterol therapy alone reduced tumor weight, but its association with M neutralized this effect. This result suggests that metformin counteracts the effect of Cb on tumor growth, either systemically or locally in the tumor. Further experiments need to be done to better understand the underlying mechanisms. The dosage and timing of use in this experiment did not reproduce the antitumor property of metformin reported by other researchers (13). In our previous work using the same model of cancer cachexia we found that naproxen alone could reduce tumor weight by approximately 60%, and in combination with Cb, circa 77% (22). Here, we showed that clenbuterol alone can reduce tumor weight by almost 40%, which demonstrates that both drugs have anti-tumor effects when combined.

Tumor cells are known to present hypermetabolism, consuming glucose at high rates through the Warburg effect (upregulation of glycolysis even in the presence of oxygen, forming lactate, which is an important product for microenvironment symbiosis and metastasis) (36). This is consistent with our findings in the non-treated tumor-bearing animals. All of the treatments normalized glucose and lactate levels, but the combinations did not present either additive nor synergic effects. We hypothesized that M, although not inducing a significant decrease of tumor weight, could impair the metabolism of tumor cells (36), leading to the results found on plasma parameters. Also, both substances (M and Cb) have lipolytic capacities (15,37), so these effects are consistent with what is shown in Figure 3C.

The control group treated with Cb (CCb) had a decrease in hepatic glycogen content. This finding can be explained by the anabolic property of clenbuterol on muscles (32). The supply of glucose to the muscles enhanced the glycogen content of the soleus. Although the presence of the tumor significantly reduced the hepatic glycogen content, none of the treatments prevented this. On the other hand, there was an apparent increase in glycogen content of the WCb group in white and red muscles compared to the W group, but this was not statistically significant. We attributed these results to the small number of animals used in this experiment (n=4 per group).

In the search for how clenbuterol might reduce tumor mass, lipid peroxidation was increased in the WCb group compared to the non-treated group (W). The addition of metformin abolished this effect, which could explain why the WMCb group did not show reduction in tumor growth. The NP-SH and hydrogen peroxide were also increased by clenbuterol therapy. The most important NP-SH is glutathione, due to its antioxidant capacity (38). Even with its increase, the tumor cell could not deal with the amount of damage generated by the increase in hydrogen peroxide and, consequently, lipid peroxidation occurred. Our results suggested that lipid peroxidation induced cell death in tumor cells in the WCb group. Metformin is known to stimulate the transcription of genes related to antioxidant activity through the activation of the transcription factor NRF2 (nuclear factor erythroid 2-related factor 2) (39). This might explain the counteracting effects of metformin in animals treated together with clenbuterol (WMCb group) on tumor growth.

The activation of β_2_-receptors is known to stimulate NADPH (nicotinamide adenine dinucleotide phosphate) oxidase, generating reactive oxygen species (40), which could explain our results. We do not fully know how clenbuterol participates in tumor growth, because there is no report on the expression of this type of receptors in Walker 256 tumor cells. Another possibility is that immune cells in the tumor microenvironment express this type of receptor, which modulates tumor response.

Our data showed that combining these drugs had no effect on improving the individual effect of these drugs on cachectic parameters. This might be because they act in the same pathways or because the treatments alone reach a ceiling effect on the studied parameters. We are not aware of any study that has reported the antitumor activity of clenbuterol alone in a model of cancer cachexia.

We point out as a limitation of our study the absence of food intake measurement, an important feature of cancer cachexia. Also, we did not seek out any mechanistic effect of the drugs, and finally, due a rapid tumor growth, the duration of treatment might not be enough to obtain a full understanding of how the drugs work as well as the dose used.

In conclusion, metformin and clenbuterol, isolated or combined, ameliorated metabolic cachexia parameters in Walker tumor-bearing rats, but only clenbuterol reduced tumor weight, a process involving lipid peroxidation.
